# Do Epigenetic Timers Control Petal Development?

**DOI:** 10.3389/fpls.2021.709360

**Published:** 2021-07-06

**Authors:** Ruirui Huang, Tengbo Huang, Vivian F. Irish

**Affiliations:** ^1^Department of Molecular, Cellular and Developmental Biology, Yale University, New Haven, CT, United States; ^2^Guangdong Provincial Key Laboratory for Plant Epigenetics, Longhua Bioindustry and Innovation Research Institute, College of Life Sciences and Oceanography, Shenzhen University, Shenzhen, China; ^3^Department of Ecology and Evolutionary Biology, Yale University, New Haven, CT, United States

**Keywords:** petal, organogenesis, *Arabidopsis*, epigenetic regulation, histones

## Abstract

Epigenetic modifications include histone modifications and DNA methylation; such modifications can induce heritable changes in gene expression by altering DNA accessibility and chromatin structure. A number of studies have demonstrated that epigenetic factors regulate plant developmental timing in response to environmental changes. However, we still have an incomplete picture of how epigenetic factors can regulate developmental events such as organogenesis. The small number of cell types and the relatively simple developmental progression required to form the *Arabidopsis* petal makes it a good model to investigate the molecular mechanisms driving plant organogenesis. In this minireview, we summarize recent studies demonstrating the epigenetic control of gene expression during various developmental transitions, and how such regulatory mechanisms can potentially act in petal growth and differentiation.

## Introduction

The formation of petals, like plant organogenesis in general, occurs *via* a period of cell division followed by post-mitotic cell expansion (Powell and Lenhard, 2012) and entails precise spatiotemporal control of gene expression. The *Arabidopsis* petal is a well- studied simple laminar organ with few cell types, making it an excellent model for understanding the mechanisms underlying plant organogenesis (Irish, 2008). A number of gene regulatory networks have been identified that act early in petal development to promote cell proliferation, and a largely distinct set of genes have been shown to participate in the later phases of cell expansion (Li et al., 2016). This transition is undoubtedly regulated by a wide variety of gene products, with a number of TEOSINTE BRANCHED1/CYCLOIDEA/PCF (TCP) transcription factors likely playing a prominent role in the temporal control of cell proliferation arrest (Czesnick and Lenhard, 2015). Despite the identification of genes and regulatory pathways regulating different phases of petal growth, the mechanisms that control this developmental shift are still largely unknown. We will discuss the possibility that epigenetic mechanisms underlie the regulation of this temporal shift, particularly in the maintenance of expression of key genes controlling early petal development.

## Epigenetic Modifications and Gene Transcription

“Epigenetic” refers to heritable changes in gene expression that are not due to changes in DNA sequence (Berger et al., 2009). In eukaryotes, DNA is packaged into chromatin, which is organized into nucleosomes that contain an octamer of histone proteins wrapped by 146 bp of DNA (Hollender and Liu, 2008). Alterations in the organization of chromatin, caused by post-translational modifications of histone proteins or DNA methylation, can affect the accessibility of chromatin to the transcriptional machinery, resulting in changes of gene expression (Sims and Reinberg, 2008). Such epigenetic changes can impact DNA replication, cell proliferation and gene transcription (Suganuma and Workman, 2008). In particular, histone modifications can alter chromatin structure directly by altering chromatin accessibility or by influencing the recruitment of effector proteins (Strahl and Allis, 2000; Karlic et al., 2010).

The amino termini of the core histones in nucleosomes are substrates of covalent modifications including acetylation, methylation, ubiquitylation, phosphorylation and SUMOylation (Roguev et al., 2001). These various modifications constitute a specific “histone code” to regulate gene expression by instructing the chromatin configuration to be either “open” or “closed” (Iñiguez-Lluhí, 2006; Lee et al., 2010). For example, histone acetylation allows the chromatin to relax and provides transcription factors and RNA polymerases access to the DNA, whereas SUMOylation appears to repress gene expression through compacting the chromatin (Iñiguez-Lluhí, 2006). By contrast, the effects of methylation and ubiquitylation depend on the residues being modified and their contexts. For example, trimethylation of lysine 27 of histone H3 (H3K27me3) catalyzed by PcG (Polycomb-group) proteins is a repressive histone modification mark, whereas methylation of lysine 4 of histone H3 (H3K4me) is an active histone modification in plants (Zhang et al., 2009).

Histone acetylation is widely studied and of particular importance to plant development, defense and adaptation (Hollender and Liu, 2008). Histone acetyltransferases (HATs) and histone deacetylases (HDACs) are enzymes required to catalyze histone acetylation and deacetylation, respectively. Many of the HATs and HDACs are components of large multisubunit complexes, which are recruited to gene promoters by DNA-bound proteins (Howe et al., 2001). For example, HDA19, a member of the RPD3/HDA1 family of HDACs, interacts with the TOPLESS (TPL) co-repressor complex and is recruited by transcription factors to specific sites on the DNA to repress gene transcription (Krogan et al., 2012; Wang et al., 2013; Oh et al., 2014). Characterization of HDAC mutants in *Arabidopsis* has indicated that the members of the RPD3/HDA1 family of HDACs play a vital role in regulating gene expression in various biological processes (Liu et al., 2014). For instance, HDA19 was shown to repress the transcription of *AGAMOUS* in the outer whorl floral organs (Krogan et al., 2012), and to repress the expression of *CIRCADIAN CLOCK ASSOCIATED 1* (*CCA1*) in controlling the circadian period (Wang et al., 2013); both processes relying on the interaction of HDA19 with the TPL co-repressor.

## Epigenetic Memory and Plant Developmental Timing

**FIGURE 1:**
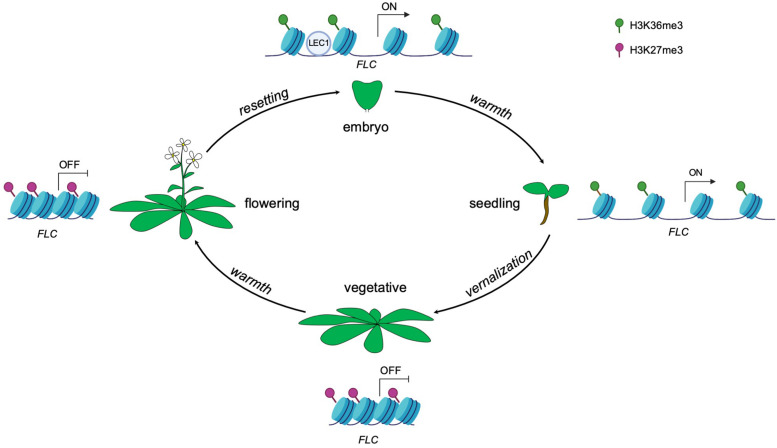
Epigenetic control of *FLC* expression by the seed-specific LEC1 protein throughout the *Arabidopsis* life cycle. Vernalization induces the deposition of the repressive histone modification mark H3K27me3, resulting in a silenced state of *FLC*. The repressive state is maintained even upon returning to warmth. The transcriptional status of *FLC* is reset in each generation during embryogenesis, resulting in the activation of *FLC*. The seed specific transcription factor LEC1 initially activates *FLC* expression by establishment of active histone modification H3K36me3 in early embryogenesis and this active memory is maintained and passed on to post-embryonic stages (seedlings).

Plants can alter gene expression in response to environmental cues and such changes in gene expression can be maintained by epigenetic memory. Once the particular state of the cell is established, the epigenetic marks can act as codes to impart expression information from the mother cell to the daughter cell and even from generation to generation (D’Urso and Brickner, 2014). In *Arabidopsis*, cold exposure (vernalization) triggers epigenetic silencing of the floral repressor *FLOWERING LOCUS C (FLC)* and in turn makes the plants competent to flower (Angel et al., 2011). The silencing of *FLC* is achieved by the replacement of active histone modifications with repressive histone modifications across the *FLC* locus. Specifically, active histone modifications such as trimethylation of lysine 36 on histone H3 (H3K36me3) across the gene body are replaced by the repressive histone modification mark H3K27me3 (Berry and Dean, 2015; Tao et al., 2017). Upon returning to warmth, *FLC* repression is epigenetically maintained at the reproductive stage including in sperm and egg cells (Berry and Dean, 2015; Figure 1). Epigenetic marks that accumulate at the *FLC* locus during vernalization need to be reset to ensure proper development of the next generation (Feng et al., 2010; Moazed, 2011). The seed specific transcription factor LEAFY COTYLEDONI (LEC1) *de novo* activates gene expression of *FLC* in the pre-embryo stage by reversing the silenced chromatin inherited from gametes to an active state (Tao et al., 2017). Interestingly, the LEC1-induced active epigenetic memory on *FLC* can be transmitted to post-embryonic stages, even well after LEC1 expression dissipates (Tao et al., 2017; Figure 1). These observations indicate that the epigenetic memory of an initial transcriptional state can be maintained for some time during plant development.

**FIGURE 2:**
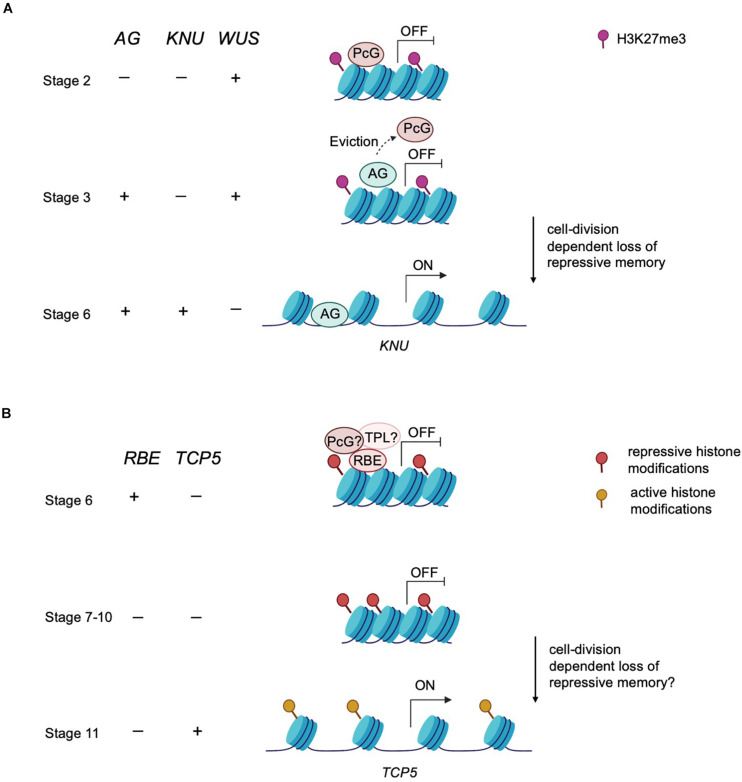
An epigenetic timer regulates floral development. **(A)** Epigenetic control of meristem determinacy. Prior to and during stage 2, repressive H3K27me3 modification catalyzed by the PcG protein is deposited across the *KNU* locus to silence *KNU* expression. At these early stages, *WUS* is expressed and maintains floral meristem stem cell proliferation. At stage 3, AG binds to the *KNU* promoter, evicting the PcG protein from *KNU*. The eviction of PcG protein induces cell division-dependent loss of H3K27me3 on *KNU*, which takes about 2 days. By stage 6, *KNU* is expressed and acts to repress *WUS* transcription and stem cell activity is then terminated. **(B)** Postulated model of epigenetic control of petal development. RBE confers repression on *TCP5* to maintain cell proliferation at early stages of petal development. RBE could repress *TCP5* transcription by recruiting epigenetic modifiers such as TPL or PcG proteins to establish repressive histone modifications across the *TCP5* locus. This repressive memory is proposed to be maintained until about stage 10. By stage 11, *TCP5* transcription is initiated, which is presumed to result from accrual of active histone modifications.

Epigenetic modifiers also regulate the timing of floral meristem development. In floral meristems, the balance between the rates of stem cell proliferation and differentiation ensures a specific size and number of floral organs (sepals, petals, stamens and carpels) (Sun et al., 2009). In *Arabidopsis*, stem cell identity is maintained by *WUSCHEL (WUS)*, whereas the termination of stem cell identity is achieved by zinc-finger protein KNUCKLES (KNU) induced repression of *WUS* (Sun et al., 2009). *KNU* is transcriptionally activated by the floral homeotic protein AGAMOUS (AG) (Figure 2A). In turn, the timing of the initiation of AG expression depends on the upregulation of the LEAFY transcription factor, which is itself upregulated in response to environmental signals that induce a feedforward loop to maintain the floral transition (Parcy et al., 1998; Busch et al., 1999; Jaeger et al., 2013). The timing of this activation is key in balancing stem cell proliferation and differentiation during floral organogenesis. The action of AG in inducing *KNU* expression requires about 2 days during which the repressive histone modification mark H3K27me3 across the *KNU* locus is removed (Sun et al., 2009). Furthermore, AG binding to the *KNU* promoter displaces PcG proteins from the locus and lead to cell division-dependent induction of *KNU* expression and further repression of *WUS* (Sun et al., 2014; Figure 2A). In sum, environmental signals induce a cascade of molecular events that culminate in the precisely orchestrated temporal transition from indeterminate to determinate growth.

## Epigenetic Control of Petal Organogenesis

After petal primordia initiate, there is a period of cell division followed by a transition to cell expansion and cell differentiation to develop to mature petals (Krizek and Fletcher, 2005). The *RABBIT EARS* (*RBE*) gene encodes a C2H2 zinc finger transcription factor containing an EAR (ERF-associated amphiphilic repression) domain and regulates petal development in *Arabidopsis*. Loss of function *rbe* mutants exhibit underdeveloped petals and absence of petal initiation (Takeda et al., 2011). These strong petal defects make *rbe* an excellent genetic tool to disclose the molecular mechanisms underlying petal development. The *TCP5* gene is a major downstream target of RBE as *tcp5-1* largely rescues the petal defects of *rbe-1* and RBE directly binds to the *TCP5* regulatory region to repress its transcription (Huang and Irish, 2015). *RBE* transcripts are localized in the presumptive petal primordia from the stage 3 to stage 6 and act to promote cell division and establish enough cells with petal identity. This specific expression pattern of *RBE* implies that the alleviation of *TCP5* repression by *RBE* commences at stage 6 (Huang and Irish, 2015). However, *TCP5* transcription is only detectable from stage 11 on during petal development, which suggests a delay of approximately 6 days between alleviation of RBE-mediated repression and activation of *TCP5* expression.

It is possible that RBE represses *TCP5* by inducing chromatin-mediated silencing at that locus. The delay of transcriptional activation of *TCP5* can be explained by the gradual elimination of this chromatin-mediated silencing, which may accompany the transition from cell division to cell expansion during petal development. It has been demonstrated that EAR motif containing proteins such as RBE recruit the TPL-HDA19 co-repressor complex to regulate gene transcription (Kagale and Rozwadowski, 2011). For instance, the EAR-motif containing *APETALA2 (AP2)* gene product recruits both TPL and HDA19 to repress the expression of multiple floral organ identity genes (Krogan et al., 2012). Additionally, EAR motif containing proteins can also interact with PcG proteins. *SUPERMAN (SUP)*, encoding a similar C2H2 zinc finger protein to RBE, can interact with *CURLY LEAF (CLF)*, a PRC2 component, to regulate floral whorl boundaries by controlling the expression of auxin biosynthetic genes (Xu et al., 2018). Furthermore, recent reports indicate that the EAR motif could act as a docking point for the TPL-HDAC and PRC2 complexes which in turn can result in chromatin remodeling at a target locus by increasing H3K27me3 and decreasing H3ac levels (Baile et al., 2020; Pelayo et al., 2021). Thus, EAR motif containing transcription factors might employ multiple layers of epigenetic modifications to maintain long-term repression.

Based on these observations, it is likely that RBE physically interacts with the TOPLESS-HDA19 complex and/or PcG proteins to temporally regulate the expression of *TCP5* during petal development. Furthermore, TOPLESS and HDA19 have been shown to act in maintaining the balance of cell division and cell differentiation. For instance, the product of the *WUSCHEL HOMEOBOX 5 (WOX5)* gene maintains the root meristem niche and prevents cell type differentiation by recruiting TOPLESS and HDA19 to repress *CYCLING DOF FACTOR 4* (*CDF4*) (Pi et al., 2015). Taken together, these results suggest RBE might orchestrate petal cell division and cell expansion by recruiting chromatin modifiers such as TPL or PcG proteins to temporally regulate *TCP5* transcription (Figure 2B). Given the role of RBE in orchestrating many aspects of petal development, this presumptive epigenetic timing mechanism could potentially function to regulate the temporal control of expression of many downstream target genes involved in petal differentiation.

## Conclusion and Perspective

To our knowledge, epigenetic control mechanisms that regulate aspects of plant development are triggered in response to external cues such as daylength or temperature. We propose here that developmental timing can also occur through a gating mechanism that depends on protein half-life. In other words, the decay of RBE protein over time could function as a timer to alter the epigenetic status of target genes such as *TCP5*, and potentially other target genes as well. It is also possible that other petal regulators containing the EAR motif, such as JAGGED (JAG), might also function by recruiting epigenetic factors to confer temporal control of petal organogenesis (Ohno, 2004; Schiessl et al., 2014). In summary, the temporal control of petal development, and more broadly organ development, may rely in part on the stochastic process of alleviating repressive epigenetic marks over time to control the transition from cell division to post-mitotic cell expansion.

## Author Contributions

RH, TH, and VFI were contributed to the writing of this review. All authors contributed to the article and approved the submitted version.

## Conflict of Interest

The authors declare that the research was conducted in the absence of any commercial or financial relationships that could be construed as a potential conflict of interest.
